# Supplementary material for: Three Ecological Models to Evaluate the Effectiveness of Trichoderma spp. for Suppressing Aflatoxigenic Aspergillus flavus and Aspergillus parasiticus
Source: Toxins (Basel). 2024 Jul 12;16(7):314. doi: 10.3390/toxins16070314 (PMC11281256; doi:10.3390/toxins16070314)
Supplement: Supplementary file 1 [file toxins-16-00314-s001.zip › toxins-3035150-supplementary.pdf]

Supplementary Material

Table 1 Strain designation, isolation information, and source of *Trichoderma* and *Aspergillus* used in this study

Strain	Designation	Isolation information	Source
Aspergillus flavus	NRRL 3357	Peanut	NRRL Agricultural Research Service (ARS)
Aspergillus parasiticus	B62	B62 derived from SU-1 (ATCC 56775)	ATCC 24690
Trichoderma asperellum	OSK-38 (*ICC-012)*	Isolated from BIO-TAM 2.0 (Isagro, Morrisville, NC)	Department of Plant Pathology (PSU)
Trichoderma asperellum	BTU	Soil	Culture collection of BTU Biotech company
Trichoderma atroviride	PNB 12-IAI	Ecuador	Department of Plant Pathology (PSU)
Trichoderma harzianum	OSK-21	Mushroom Spawn Lab	Department of Plant Pathology (PSU)
Trichoderma harzianum	OSK-34	Isolation from Custom GP (Custom biologicals, Boca Raton, FL)	Department of Plant Pathology (PSU)
Trichoderma virens	OSK-13	Isolated from Rootshield Plus+ (BioWorks, Victor, NY)	Department of Plant Pathology (PSU)
Trichoderma virens	OSK-36	Isolation from Custom GP (Custom biologicals, Boca Raton, FL)	Department of Plant Pathology (PSU)
Trichoderma viride	OSK-22 (208)	Mushroom Spawn Lab	Department of Plant Pathology (PSU)
